# The independent and interactive effects of changes in overtime and night shifts during the COVID-19 pandemic on burnout among nurses: a longitudinal study

**DOI:** 10.5271/sjweh.4176

**Published:** 2024-09-01

**Authors:** Emanuele Maria Giusti, Giovanni Veronesi, Francesco Gianfagna, Nicola Magnavita, Francesca Campana, Rossana Borchini, Licia Iacoviello, Marco Mario Ferrario

**Affiliations:** 1 EPIMED Research Center, Department of Medicine and Surgery, University of Insubria, Varese, Italy.; 2 Mediterranea Cardiocentro, Napoli, Italy.; 3 Department of Life Sciences and Public Health, Università Cattolica del Sacro Cuore, Rome, Italy.; 4 ASST Sette Laghi, Varese, Italy.; 5 ASST Lariana, Como, Italy.; 6 Department of Medicine and Surgery, LUM University, Casamassima, Italy.; 7 Department of Epidemiology and Prevention, IRCCS Neuromed, Pozzilli, Italy.

**Keywords:** Work stressors, healthcare workers, occupational health, shift work, mental health, long working hours

## Abstract

**Objectives:**

This study aimed to evaluate the independent and interactive effects of changes in overtime and night shifts on burnout among nurses during the COVID-19 pandemic.

**Methods:**

Nurses working in an Italian university hospital (N=317) completed the Maslach Burnout Inventory in September 2019 and again in December 2020. Based on hospital administrative data, changes in overtime and night shifts in the same years were categorized into three groups each. Linear regressions were used to estimate 2020 burnout differences between exposure groups, controlling for 2019 burnout levels, demographic and work-related characteristics, and to test the interaction between the two exposures.

**Results:**

Nurses in the *onset of high overtime* group had higher emotional exhaustion [4.33, 95% confidence interval (CI) 1.74−6.92], depersonalization (2.10, 95% CI 0.49−3.71), and poor personal accomplishment (2.64, 95% CI 0.55−4.74) compared to *stable low overtime* nurses. Nurses in the *increase in night shifts* group had lower emotional exhaustion (-4.49, 95% CI -7.46− -1.52) compared to *no night shift* nurses. Interaction analyses revealed that this apparently paradoxical effect was limited to *stable low overtime* nurses only. Moreover, increases in night shifts were associated with higher depersonalization and poor personal accomplishment in nurses in the *stable high overtime* group.

**Conclusions:**

Increase in overtime is an independent risk factor for burnout among nurses, highlighting the need for specific regulations and actions to address it. Long-standing guidelines for the assignment of night shifts might have contributed to attenuate the impact of their increase on nurses’ mental health.

Burnout has been defined as a psychological syndrome encompassing feelings of emotional exhaustion, cynicism related to one’s job, and a sense of lack of personal accomplishment (1). Burnout is rooted in the workplace and has been linked to the interplay between work-related conditions and individual resources, with different theories elucidating their relative significance (2). Nurses, including nurse assistants, exhibit a notable prevalence of burnout, with an estimated prevalence of 11% before the COVID-19 pandemic (3). During previous epidemic outbreaks, a high prevalence of burnout was observed in this population, likely due to the organizational problems that occur during such periods (4). During the pandemic, studies evaluating its prevalence suggested that up to half of nurses were affected (4, 5), in particular among those caring for COVID-19 patients (6), surpassing the rates observed in other healthcare workers (7). This had far-reaching consequences since burnout has a detrimental impact on the physical and psychological health of the sufferer and causes organizational issues, including work inefficiencies, elevated turnover rates and, among nurses, an increase in errors and decreased quality of care (8–10). Understanding how work-related conditions impact burnout is therefore important both for safeguarding the health of nurses and for improving the capacity of healthcare systems to face crises.

Multiple factors related to the organization of work have been identified as predictors of burnout, including overtime and shift work (11–14). Overtime has been related to burnout through different mechanisms that both increase the exposure to work-related stressors and reduce the resources available to manage them. Working overtime implies longer durations of effort and increased contact with workplace dysfunctions, potentially increasing perceived stress and, consequently, leading to burnout (15). The depletion of resources stems from the fact that overtime not only increases fatigue, but also diminishes the time available to recover from it. In addition, overtime reduces sleep quality impairs cognitive functioning, and increases work-family conflicts (16–19). These aspects have gained considerable importance during the pandemic as the need to face the crisis led to a surge in the demand of work from nurses, in particular in COVID-19 wards (15, 20). Similar to overtime, shift work is associated with burnout due to increased exposure to work stress and depletion of resources. Specifically, night shifts heighten the perception of more demanding or constraining working conditions, reduce sleep quality and increase the risk of sleep disorders (21–23). It has been suggested that these factors, when concurrently present, might exert a combined effect, making it important to assess the effect of their interaction (24). Cross-sectional studies performed during the pandemic showed that increased night shifts and overtime were associated with perceived stress among nurses (25–27). Nonetheless, the paucity of longitudinal investigations into these factors, both before and during the pandemic, hampers the evaluation of how night shifts and overtime, as well as their change, might affect burnout over time. Furthermore, numerous studies rely on self-reported measures of overtime and night shifts, which are susceptible to recall bias, whereas administrative data may offer a more accurate depiction (28, 29).

The aim of this longitudinal study was to evaluate – using administrative data on changes in overtime and night shifts from a sample of Italian nurses investigated before and during the COVID-19 pandemic – the independent associations and the interactive effects of these changes on burnout, controlling for its pre-pandemic levels. Previous results from the same study showed that pre-pandemic perceived work stressors and work satisfaction were associated with the transition to burnout among nurses (11).

## Methods

The *North Italian Longitudinal Study Assessing the Mental Health Effects of SARS-CoV-2 Pandemic in Health Care Workers* is a longitudinal study conducted in a university hospital in the city of Varese, Italy, comprising two surveys targeting healthcare workers, the first in September 2019, before the pandemic outbreak, and the second in December 2020 during the second wave. Extensive details about the study design and measurement procedures can be found in a previously published paper (30). In brief, in each survey, participants completed a questionnaire measuring demographic (age, sex) and work-related characteristics (full- or part-time work), and several mental health variables, including burnout. In the second survey, the participants were also asked if they had worked in a COVID-19 ward in 2020. For the first survey, 844 nurses and nurse assistants were invited, and 599 (71%) agreed to participate. In December 2020, we contacted the 541 nurses still at work, 346 of whom participated in the second survey (64%). There were no differences among nurses who responded to the first survey only or to both surveys in terms of age, sex, full-time work, number of night shifts, overtime hours and pre-pandemic burnout (supplementary material, www.sjweh.fi/article/4176, table S1). From the 346 nurses who participated in both study surveys, we excluded 21 workers with ≥150 days of absence in either 2019 or 2020 as recorded in the payroll archives. Excluded nurses were similar in age, sex and level of pre-pandemic burnout compared to included nurses (all P>0.05, data not shown). Furthermore, we excluded 8 nurses with missing burnout data in 2019 or 2020, leaving a final sample size of 317 nurses and nurses assistants.

### Assessment of burnout

Burnout was measured in both surveys using the Maslach Burnout Inventory (MBI) (31), a self-report questionnaire investigating the frequency of symptoms related to emotional exhaustion, depersonalization, and poor personal accomplishment. We calculated the scores of its refined version, which is based on a previous study investigating its psychometric properties among healthcare workers (32). This refined version includes 18 items from the original questionnaire, 6 items measuring emotional exhaustion, 5 items measuring depersonalization, and 7 items measuring poor personal accomplishment. All the items are on a Likert scale ranging from 0 (“never”) to 6 (“every day”), with higher scores indicating higher levels of the respective burnout dimension. The refined version showed adequate internal consistency, structural validity and longitudinal invariance (32).

### Definition of the exposure variables

Based on individual consent, for each participant, we accessed the hospital payroll archives for the years 2019 and 2020, with yearly individual-level information on the number of worked days, the number of work hours due by contract, and the number of effective worked hours, eg, excluding non-working periods due to sickness absences, injuries, maternity leaves, and holiday periods. In addition, we accessed the hospital administrative dataset with the individual-level daily shift schedule, from 1 January 2019 to 31 December 2020. Based on European regulations, night work is defined as working ≥3 hours in the period 24:00–05:00 hours (33). During the pandemic, the regular 8-hour night shifts were extended to 12 hours, provided that workers were in good health and did not have any clinical limitations. Each participant was linked to their administrative record, using a unique ID code, to retrieve data for the calculation of the overtime and night shift indicators. Overtime was operationalized as contractual overtime, ie, working for longer than the stated in the contract (34). In particular, we calculated the annual average of daily overtime (in hours) as the difference between the hours worked per day (ratio of the number of hours worked during the year and the number of working days in the year) and the daily hours due by contract. As an indicator of night shifts, we retrieved the number of scheduled night shifts per year.

The exposure variables for the present work are 'change in daily overtime' and 'change in the number of night shifts' between 2019 and 2020, defined based on the distributions of both variables in the two years (supplementary figure S1). For the overtime change groups, we used as cut-off thresholds 1 hour in 2019 and 2 hours in 2020 to group nurses into the following categories: (i) *stable low overtime* (SLO, 53%) who were below the thresholds in both years; (ii) *stable high overtime* (SHO, 15%) who were above the 2019 threshold and continued to be exposed in 2020 at similar levels; and (iii) *onset of high overtime* (OHO, 32%) who were <1 hour in 2019 and >2 hours in 2020.

The number of night shifts per year was categorized into four classes: 0, <48, 48–59, and ≥60 night shifts. Then, we defined the change in night shifts groups as: *No night shifts* (NNS, 32%), including those who did no shift work in 2019 and 2020; *no increase in night shifts* (NINS, 39%), including those who either remained in the same class or switched to a lower class; and *increase in night shifts* (INS, 28%), including those who switched to a higher class from 2019 to 2020.

### Statistical analyses

Differences among the exposure variables regarding socio-demographic characteristics (age, sex), work-related factors (self-reported full- versus part-time work in 2019 and having worked in a COVID-19 ward in 2020), and burnout levels in 2019 were ascertained using chi-square tests or ANOVAs.

Then, we assessed the associations between the exposure variables and the three burnout dimensions in 2020, using nested linear regression models. First, we estimated in separate models the association between each exposure variable and each burnout dimension during the pandemic, adjusting for age, sex and having worked in a COVID-19 ward (Model 1). In a next step, we estimated a model including also both exposure variables, to elucidate their mutually independent effects (Model 2). Finally, the respective baseline burnout dimension levels in 2019 were added (Model 3), characterizing the estimates from a study with longitudinal information on both exposures and outcomes. In a sensitivity analysis, these latter models were also estimated only among nurses with a full-time contract. Finally, we investigated the interaction between change in overtime and change in night shifts, after collapsing the categories of the latter into *No night shifts or not increased* (including NNS and NINS nurses) and *Increased* (including INS nurses), due to sample size considerations. This analysis was done by adding the interaction term to Model 3 (formal test of no interaction: 2 df Wald chi-square).

We report the β coefficients with 95% confidence intervals (CI), which estimate the mean differences in the relevant burnout dimension from the reference category. The analyses were performed using SAS, version 9.4 (SAS Institute Inc, Cary, NC, USA).

## Results

Among the 317 nurses in the sample, 261 (82.3%) were female. The mean age was 46.4 [standard deviation (SD) 9.4] years, 272 (85.8%) were working full-time and 179 (56.5%) reported having worked or were still working in a COVID-19 ward.

The mean daily overtime was 0.62 (SD 0.51) hours in 2019 and 1.62 (SD 0.94) hours in 2020, with a significant mean increase of 1.00 (95% CI 0.92−1.08) hour between the two periods in the whole sample, and of 1.71 (95% CI 1.64−1.77) hours in the OHO group. In 2019, 82.7% of nurses had <1 hour of daily overtime, and only 0.6% had >2 hours of daily overtime; these figures were 32.5% and 42.0% in 2020, respectively.

The overall mean number of night shifts per year among nurses who performed ≥1 night shift in 2019 was 52.18 (SD 17.56) in 2019 and 54.64 (SD 17.79) in 2020, with a significant mean increase of 2.46 (95% CI 0.51– 4.41) between the two periods. Those in the INS group performed 35.61 (SD 21.85) night shifts in 2019 and 52.69 (SD 18.15) in 2020, with a significant mean increase of 17.09 (95% CI 14.83 – 19.33). Among nurses who were not assigned to night shifts in 2019 (N=109), 67.9% (N=74) continued to be unexposed in 2020.

Table 1 shows the socio-demographic and work-related characteristics, and burnout levels in 2019 of the enrolled nurses, stratified by the exposure variables. Nurses in the OHO, NINS and INS groups were more likely to be male, younger, and to have worked in a COVID-19 ward compared to nurses in the reference, non-exposed categories (SLO and NNS, respectively). The age- and gender-adjusted mean levels of the three burnout dimensions in 2019 did not differ across the exposure categories.

**Table 1:** Demographic and work-related characteristics and burnout levels at baseline (2019), stratified by exposure categories. [SD=standard deviation; SE=standard error.]

		**Change in overtime**					**Change in night shifts**			
		Stable low overtime (N=168)	Stable high overtime (N=49)	Onset of high overtime (N=100)	P-value ^a^		No night shifts (N=103)	No increase in night shifts (N=124)	Increase in night shifts (N=90)	P-value ^a^
Female sex, N (%)		146 (86.9)	44 (89.8)	71 (71.0)	0.001		95 (92.2)	100 (80.7)	66 (73.3)	0.002
Age, mean (SD)		48.8 (8.8)	45.4 (9.9)	42.9 (8.9)	<0.001		49.8 (8.2)	46.4 (9.4)	42.6 (9.2)	<0.001
Full-time work in 2019, N (%)		146 (86.9)	40 (81.6)	86 (86.0)	0.44		89 (86.4)	102 (82.3)	81 (90.0)	0.20
Worked in a COVID-19 ward, N (%)		82 (48.8)	32 (65.3)	65 (65.0)	0.01		35 (34.0)	82 (66.1)	62 (68.9)	<0.001
Emotional exhaustion in 2019, mean (SE) ^b^		20.1 (0.7)	18.8 (1.3)	20.4 (0.9)	0.58		20.7 (0.9)	19.7 (0.8)	19.5 (1.0)	0.65
Depersonalization in 2019, mean (SE) ^b^		10.0 (0.4)	10.1 (0.7)	10.9 (0.5)	0.43		9.5 (0.5)	10.5 (0.5)	10.9 (0.6)	0.15
Poor personal accomplishment in 2019, mean (SE) ^b^		20.3 (0.69)	20.3 (1.2)	21.1 (0.9)	0.76		19.6 (0.9)	20.0 (0.8)	22.4 (0.9)	0.07
	Change in overtime									
	Stable low overtime, N (%)	-	-	-	-		98 (95.2)	39 (31.5)	31 (34.4)	<0.001
	Stable high overtime, N (%)	-	-	-			3 (2.9)	36 (29.0)	10 (11.1)	
	Onset of high overtime, N (%)	-	-	-			2 (1.9)	49 (39.5)	49 (54.4)	

^a^ Chi-square tests for categorical variables or t-tests for continuous variables comparing groups based on the relevant exposure variable. ^b^ Age- and gender-adjusted means.

**Figure 1:**
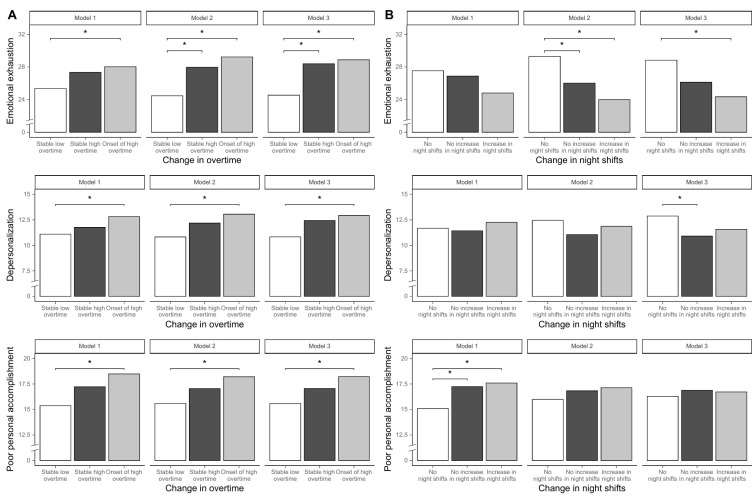
Mean burnout levels across exposure categories, adjusting for covariates (Model 1), mutual exposures (Model 2), and pre-pandemic burnout levels (Model 3). Note. Panel A displays mean burnout levels across change in overtime categories, Panel B across change in night shifts categories. Model 1: adjusted for age, sex, and having worked in a COVID-19 ward. Model 2: Model 1 + the other exposure. Model 3: Model 2 + the relevant burnout dimension in 2019.

Table 2 shows the associations between change in overtime and burnout. Compared to nurses in the SLO group (reference), nurses in the OHO group had higher means of all three burnout dimensions, when controlling for age, sex and working in COVID wards (Model 1). When further adjusting for change in night shifts (Model 2), the differences between OHO and SLO categories increased: 4.77 (95% CI 1.93−7.60) for emotional exhaustion, 2.24 (95% CI 0.42−4.06) for depersonalization and 2.62 (95% CI 0.47−4.77) for poor personal accomplishment. In addition, the differences in emotional exhaustion between SHO and SLO nurses became statistically significant (95% CI 3.50, 0.13−6.87). Further adjustment for baseline burnout levels (Model 3) slightly modified the above-described differences, with statistically significant overall Wald tests for all three MBI dimensions. The adjusted means based on these three models are reported in figure 1, Panel A. These associations were similar when the analyses were restricted to full-time nurses only (supplementary table S2).

**Table 2:** Differences in the mean levels of the three burnout dimensions between change in overtime groups.

		Model 1 ^a^			Model 2 ^b^			Model 3 ^c^	
		β^d^ (95% CI)	P-value ^e^		β^d^ (95% CI)	P-value ^e^		β^d^ (95% CI)	P-value ^e^
Emotional exhaustion									
	Stable low overtime	Reference	0.09		Reference	0.003		Reference	0.002
	Stable high overtime	1.97 (-1.10–5.05)			3.50 (0.13–6.87)			3.84 (0.77–6.92)	
	Onset of high overtime	2.66 (0.17–5.16)			4.77 (1.93–7.60)			4.33 (1.74–6.92)	
Depersonalization									
	Stable low overtime	Reference	0.10		Reference	0.05		Reference	0.03
	Stable high overtime	0.67 (-1.28 –2.62)			1.36 (-0.80–3.53)			1.61 (-0.31–3.52)	
	Onset of high overtime	1.72 (0.14 –3.30)			2.24 (0.42–4.06)			2.10 (0.49–3.71)	
Poor personal accomplishment									
	Stable low overtime	Reference	0.004		Reference	0.06		Reference	0.046
	Stable high overtime	1.85 (-0.45–4.15)			1.46 (-1.10–4.01)			1.48 (-1.01–3.97)	
	Onset of high overtime	3.11 (1.25–4.97)			2.62 (0.47–4.77)			2.64 (0.55–4.74)	

^a^ Adjusted for age, sex, and having worked in a COVID-19 ward. ^b^ Model 1 + change in night shifts. ^c^ Model 2 + the relevant burnout dimension in 2019. ^d^ Multivariable-adjusted mean differences (β) compared to the reference categories. ^e^ Wald chi-square test (2 degrees of freedom).

As reported in table 3, nurses in the INS and NINS groups, when controlling for age, sex, and work in a COVID-ward (Model 1), did not differ in terms of emotional exhaustion and depersonalization compared to NNS nurses but showed higher poor personal accomplishment. When further adjusting for concurrent change in overtime (Model 2), the differences in poor personal accomplishment among these categories reduced drastically; while differences in emotional exhaustion became statistically significant (INS versus NNS: -5.30; 95% CI -8.55− -2.04, NINS versus NNS -3.27; 95% CI -6.31− -0.23), remaining so after adjusting for the baseline levels of the relevant burnout dimension (Model 3) in the INS group (-4.49; 95% CI -7.46− -1.52). The same effect became statistically significant for depersonalization among nurses in the NINS group (-1.97; 95% CI -3.70− -0.23), although the overall Wald test was not significant (P=0.08). Poor personal accomplishment differences among change in night shifts groups further decreased in Model 3. Similar results were obtained when the analyses were restricted to full-time nurses only (supplementary table S2), and after stratification for hospital department and working in a COVID-19 ward (data not shown). The adjusted means of the change in night shifts groups based on Models 1-3 are reported in Figure 1, Panel B.

**Table 3:** Differences in the mean levels of the three burnout dimensions between change in night shifts groups.

		Model 1 ^a^			Model 2 ^b^			Model 3 ^c^	
		β^d^ (95% CI)	P-value ^e^		β^d^ (95% CI)	P-value ^e^		β^d^ (95% CI)	P-value ^e^
Emotional exhaustion									
	No night shifts	Reference	0.16		Reference	0.01		Reference	0.01
	No increase in night shifts	-0.65 (-3.28–1.97)			-3.27 (-6.31– -0.23)			-2.70 (-5.48–0.07)	
	Increase in night shifts	-2.73 (-5.67–0.21)			-5.30 (-8.55– -2.04)			-4.49 (-7.46– -1.52)	
Depersonalization									
	No night shifts	Reference	0.62		Reference	0.33		Reference	0.08
	No increase in night shifts	-0.25 (-1.92–1.43)			-1.40 (-3.35–0.55)			-1.97 (-3.70– -0.23)	
	Increase in night shifts	0.58 (-1.29–2.45)			-0.60 (-2.68–1.49)			-1.30 (-3.16–0.55)	
Poor personal accomplishment									
	No night shifts	Reference	0.047		Reference	0.65		Reference	0.87
	No increase in night shifts	2.15 (0.18–4.13)			0.84 (-1.47–3.15)			0.60 (-1.65–2.84)	
	Increase in night shifts	2.51 (0.30–4.73)			1.15 (-1.32–3.62)			0.43 (-2.00–2.85)	

^a^ Adjusted for age, sex, and having worked in a COVID-19 ward. ^b^ Model 1 + change in night shifts. ^c^ Model 2 + the relevant burnout dimension in 2019. ^d^ Multivariable-adjusted mean differences (β) compared to the reference categories. ^e^ Wald chi-square test (2 degrees of freedom).

Table 4 reports the results of the model evaluating the interaction between increase in night shifts and change in overtime by presenting the mean difference in burnout levels between nurses who experienced an increase in night shifts and those who did not, across the levels of change in overtime. The interaction was significant for all three burnout dimensions (Wald chi-square p-values <0.05). In particular, increase in night shifts was associated with lower emotional exhaustion in nurses in the SLO group (-5.43, 95% CI -8.83− -2.04) and with higher depersonalization (4.34; 95% CI 0.62−8.07) and poor personal accomplishment (5.65, 95% CI 0.87−10.43) in the SHO group.

**Table 4:** Association between increase in night shift and burnout dimensions, by change in overtime groups.

Change in overtime		Increase in night shifts	Adjusted mean ^b^	β^c^ (95% CI)	Interaction test p-value ^a^
Emotional exhaustion					
	Stable low overtime	No night shifts or not increased	26.27	Reference	0.030
		Increase in night shifts	20.83	-5.43 (-8.83– -2.04)	
	Stable high overtime	No night shifts or not increased	27.35	Reference	
		Increase in night shifts	30.73	3.38 (-2.57–9.33)	
	Onset of high overtime	No night shifts or not increased	28.41	Reference	
		Increase in night shifts	27.24	-1.67 (-4.54–2.20)	
Depersonalization					
	Stable low overtime	No night shifts or not increased	11.35	Reference	0.049
		Increase in night shifts	10.34	-1.01 (-3.12–1.11)	
	Stable high overtime	No night shifts or not increased	11.11	Reference	
		Increase in night shifts	15.45	4.34 (0.62–8.07)	
	Onset of high overtime	No night shifts or not increased	12.53	Reference	
		Increase in night shifts	12.63	0.10 (-2.01–2.20)	
Poor personal accomplishment					
	Stable low overtime	No night shifts or not increased	15.58	Reference	0.046
		Increase in night shifts	14.84	-0.74 (-3.5–2.02)	
	Stable high overtime	No night shifts or not increased	16.08	Reference	
		Increase in night shifts	21.72	5.65 (0.87–10.43)	
	Onset of high overtime	No night shifts or not increased	18.85	Reference	
		Increase in night shifts	17.85	-0.99 (-3.70–1.71)	

^a^ Wald chi-square tests (2 degrees of freedom). ^b^ Mean level from the regression model at marginal means of the covariates. ^c^ Mean difference compared to the reference category, from linear regression models including age, sex, having worked in a COVID-19 ward, both exposure variables and their interactions, and the levels of the corresponding burnout dimension in 2019.

## Discussion

This longitudinal study aimed to assess burnout among nurses during the COVID-19 pandemic in relation to changes in overtime and night shifts controlling for pre-pandemic burnout levels, and elucidating the independent effects of each exposure, as well as their interaction. Our findings showed that increases in overtime were associated with higher scores in all three dimensions of burnout, while change in night shifts had modest overall effects on poor personal accomplishment and depersonalization but also an apparent paradoxical effect on pandemic emotional exhaustion. Finally, the presence of an interaction suggests that the effect of the increase in night shifts was dependent on the level of change in overtime.

Compared to nurses in the SLO group, emotional exhaustion was higher in the SHO and OHO groups, the latter being characterized by higher levels of depersonalization and poor personal accomplishment. These effects emerged more clearly when controlling for concurrent change in night shifts and baseline levels of burnout and were observed also when the analysis was restricted to full-time nurses. This confirms previous findings mainly from cross-sectional studies (24, 26, 35–37) and shows that these effects are independent from previous burnout levels, and other variables, including night shifts. The higher emotional exhaustion of SHO compared to SLO nurses might be related to the chronic exposure to high workload, which might increase conflicts between work schedule and private life (38). The highest levels of the three dimensions of burnout in OHO nurses could be attributed to the profound impact of the pandemic on their professional and personal lives. Considering that, for nurses with a full-time contract of 38 hours per week, two daily overtime hours correspond to 48 hours per week, which is the maximum permitted overtime in the EU (33), these nurses had a major surge in their worktime, likely disrupting their previous routine. We recommend the implementation of other longitudinal studies to confirm this result since, to our knowledge, the only longitudinal study on this topic, carried out on Taiwanese healthcare workers, did not report stratified results for different health professionals and did not consider baseline levels of overtime (7).

The relationship between change in night shift and burnout dimensions was more complex. The association between the increase in night shift and poor personal accomplishment was not independent from individual and work-related characteristics and therefore suggests a potential spurious association, already identified in a cross-sectional national survey of Malaysian nurses (39). We did not find higher levels of exhaustion and depersonalization for nurses in stable or increased number of night shifts categories when compared to not exposed nurses, controlling for age, gender, and work in COVID-wards. After adjusting for concurrent change in overtime and baseline burnout levels, we found two apparently paradoxical effects: compared to nurses in the NNS group, nurses who increased the number of night shifts during the pandemic showed lower levels of emotional exhaustion, while those who did not increase night shifts showed lower levels of depersonalization. The interaction analyses revealed that the former effect was driven by SLO nurses; and that SHO nurses who increased the frequency of night shifts had higher levels of poor personal accomplishment and depersonalization. All things considered, our findings suggest that an increase in the number of night shifts is not detrimental per se, and that it might become so under conditions of chronically high overtime. Previous studies on nurses were mainly cross-sectional, the large majority investigating the pandemic levels of night shifts, and only in a few cases the self-reported change in workload (7, 24–27, 37, 39). These methodological issues hamper the comparison with our results. In general, we notice that the reported associations between night shifts and burnout are poor and mixed and are the strongest in populations with concomitant exposure to high levels of overtime (37, 39, 40).

The lower levels of emotional exhaustion among those who increased their night shifts, which appeared only when controlling for concomitant overtime as well as for pre-pandemic emotional exhaustion levels, merits more in-depth consideration. The apparently paradoxical effect might be explained assuming by a selection process which took place during the pandemic for assigning work schedules. In our study, nurses with increases in night shifts and overtime were more likely to be younger and male and the large majority (67.9%) of nurses who were not assigned to night shifts in 2019 continued to be unexposed in 2020. In addition, on average the increase corresponded to one additional night shift every three weeks among nurses who, before the pandemic, engaged in a mean of 35 night shifts per year. The assignment of nurses to night shifts in Italy and most European countries is regulated by well-established safety rules, based on individual work-fitness and attitudes to adapt to changes in the circadian rhythm, and the absence of work limitations that are repeatedly assessed at regular intervals (33, 41). The guidelines for assigning overtime are less established as existing regulations do not provide specific rules for preventing overtime in cases where it may not be advisable and just specify a maximum amount of overtime, ie, 48 hours of work per week based on Italian and European regulations and up to 250 hours per year based on the relevant Italian collective labor agreement (33). The shortage of personnel during the pandemic likely exacerbated this exposure, leading to detrimental effects on burnout.

Previous studies found age and sex differences in the allocation of night shifts and overtime hours; this might be related to differences in the stages of the career since nurses tend to move from shift work to daywork as they progress (23, 42). Sex differences in the distribution of overtime have been previously reported in a study on Canadian nurses from 2005 to 2008 that found that male nurses, full time nurses, and nurses working in emergency, general medicine and intensive care units, worked more overtime hours (43). These differences might be related to differing familial roles, since those more involved in childcare tend to work less overtime (44). It is therefore possible that nurses who worked the most overtime might be those with the lowest degree of choice (45), in contrast with the current European legislation (33). Therefore, since the uneven distribution of change in overtime resulted in increased burnout outcomes among nurses, it is important to proactively plan these changes to mitigate such inequality.

The primary strength of this study is inherent in its longitudinal design, with the same individuals investigated twice, which enabled the examination of changes in overtime and night shifts and their impact on burnout accounting for its initial levels. Secondly, self-selection bias is unlikely given the satisfactory participation rates at both surveys (30). Thirdly, the use of administrative data to assess the exposures overcame the limitations of self-report data, which can be susceptible to recall and social desirability biases. A limitation of our study is the fact that we assessed the number of scheduled night shifts instead of their actual number. However, scheduled night shifts are updated in case of sickness. When nurses exchange their shifts for other reasons, the total number of scheduled night shifts is likely to closely resemble the actual number on an annual base. Another limitation concerns the sample size, which was not large enough to allow a more detailed classification of both exposures to investigate dose-response relationships. Finally, our study focused on organizational changes resulting from the pandemic, therefore we are not able to disentangle the effects of changes from the fact that these changes occurred during a pandemic period. However, research has shown that organizational changes can contribute to perceived stress also outside healthcare crises, in particular if workers have little control over them (46, 47). Stemming from our results, further investigations are warranted to assess whether changes in night shifts and overtime are associated with burnout also in those instances.

### Concluding remarks

Our study showed the complex relationships between changes in work organization conditions and burnout among nurses, highlighting the importance of assessing changes in exposures and outcomes rather than focusing solely on their final levels. The onset of high overtime, being exposed to chronically high overtime, and increasing night shifts when already enduring high overtime are major independent risk factors for burnout. The presence of long-standing guidelines for the assignment of night shifts, based on scientific evidence on health effects of changes in circadian rhythm, might have contributed to attenuate the impact of the increase in night shifts on the mental health of nurses. The results of this study highlight the need of more robust and innovative guidelines on managing overtime hours aimed at safeguarding nurses’ mental health, which should take into consideration work-related stress conditions and a healthy work-life balance in addition to limitations based on clinical assessments. Further studies are needed to investigate the effect of work scheduling regulations in working conditions at different levels of constraints and work stress.

### Protection of research participants

This study was approved by the Research Ethics Committee of the ASST Sette Laghi (approval ID: 69/2020). Prior to participation, all individuals provided their informed consent by signing a consent form. The study was conducted in accordance with the principles outlined in the Helsinki Statement.

## Supplementary material

Supplementary material
