# Supplementary material for: The independent and interactive effects of changes in overtime and night shifts during the COVID-19 pandemic on burnout among nurses: a longitudinal study
Source: Scand J Work Environ Health. 2024 Aug 30;50(6):475–84. doi: 10.5271/sjweh.4176 (PMC11395668; doi:10.5271/sjweh.4176)
Supplement: Supplementary material [file SJWEH-50-475-S001.pdf]

The independent and interactive effects of changes in overtime and night shifts during the COVID-19 pandemic on burnout among nurses: a longitudinal study[1]

Emanuele Maria Giusti, PhD, Giovanni Veronesi, PhD,[2] Francesco Gianfagna, MD, Nicola Magnavita, MD, Francesca Campana, MD, Rossana Borchini, MD, Licia Iacoviello, MD, Marco Mario Ferrario, MDm

1. Supplementary materials
2. Corresponding author: Prof. Giovanni Veronesi, EPIMED Research Center, Department of Medicine and Surgery, University of Insubria, 21100 Varese, Italy. [Email: giovanni.veronesi@uninsubria.it].

Figure S1. Distribution of daily overtime (Panel A) and annual number of night shifts (Panel B) in the studied sample of nurses.

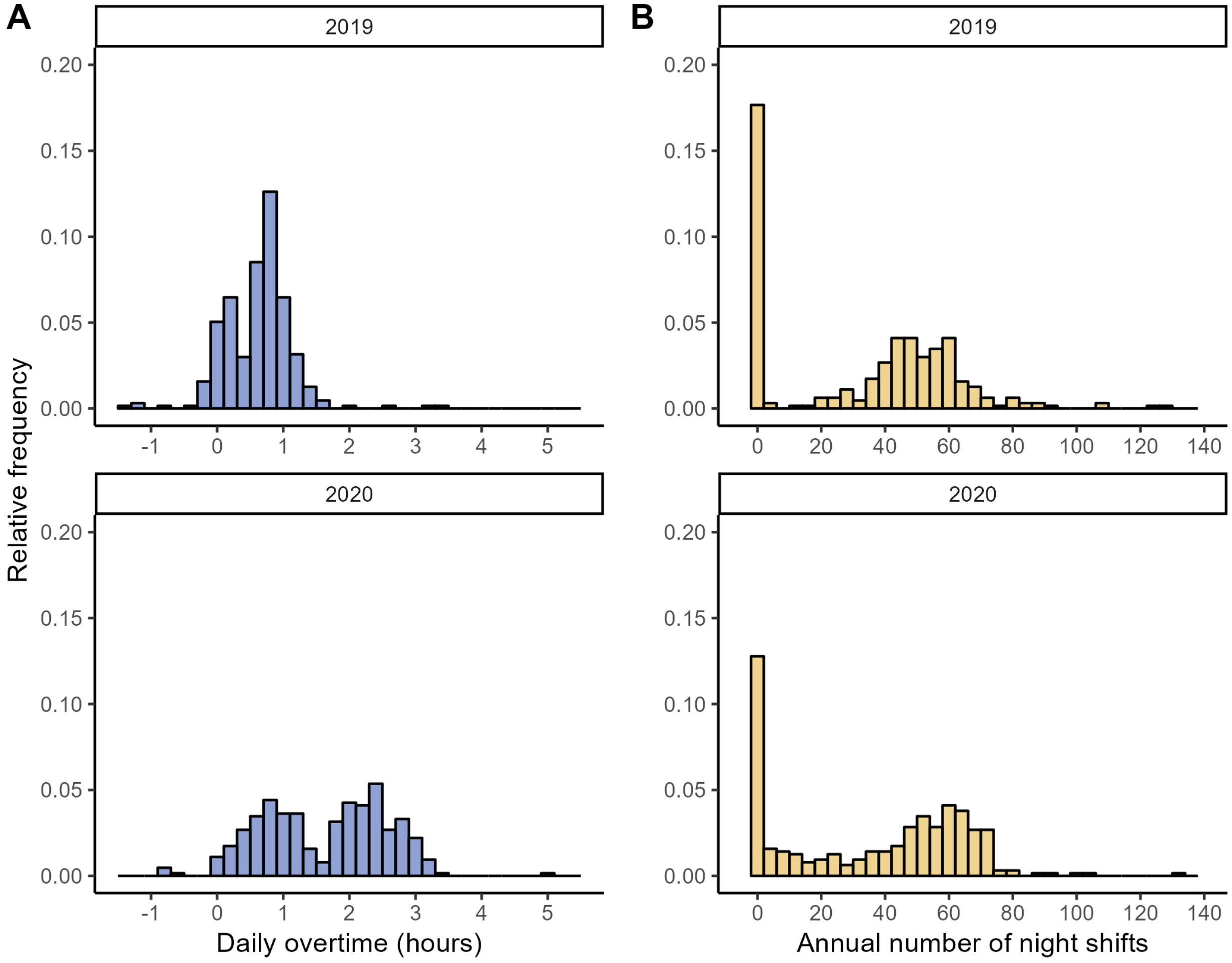

Table S1. Comparison of socio-demographic, burnout and work-related variables collected in 2019 between nurses who responded to the 2019 and 2020 survey and nurses who responded to the 2019 survey only.

	Respondents in 2019 and 2020 (n=346)	Respondents in 2019 only (n=245)	p-value[a]
Female sex, No. (%)	270 (79.0)	193 (78.8)	0.96
Age, mean (SD)	46.2 (9.5)	46.4 (10.7)	0.80
Full time work in 2019, No. (%)	296 (85.6)	218 (88.9)	0.22
Night shift work in 2019, No. (%)	215 (62.9)	139 (56.7)	0.13
Number of night shifts in 2019, mean (SD)	32.9 (29.9)	30.9 (29.0)	0.40
Number of night shifts in 2019 among night shift workers[b], mean (SD)	52.2 (16.6)	53.2 (15.9)	0.58
Extra hours per day in 2019, mean (SD)	0.6 (0.5)	0.6 (0.5)	0.39
Emotional exhaustion 2019[c], mean (SD)	19.9 (9.1)	19.8 (9.5)	0.85
Depersonalization 2019[c], mean (SD)	10.4 (5.2)	9.7 (5.0)	0.20
Poor personal accomplishment 2019[c], mean (SD)	20.4 (8.6)	21.5 (9.2)	0.17

[a] from chi-square tests for categorical variables or t-tests for continuous variables.
[b] night shift workers were 215 among respondents, and 139 among non respondents.
[c] 4 participants had missing data in these variables.

Table S2. Results of the linear regression models evaluating the mutually-adjusted effect of the exposure variables on the three burnout dimensions in full-time nurses (n=272).

	**Emotional exhaustion**	**Depersonalization**	**Poor Personal Accomplishment**
	**β[a] (95% CI)**	**β[a] (95% CI)**	**β[a] (95% CI)**
**Change in overtime**			
Stable low overtime (SLO)	Ref	Ref	Ref
Stable high overtime (SHO)	4.59 (1.08 - 8.14)	1.91 (-0.35 - 4.18)	0.88 (-1.91 - 3.67)
Onset of high overtime (OHO)	4.26 (1.40 - 7.12)	2.25 (0.43 - 4.07)	2.65 (0.40 - 4.91)
**Change in night shifts**			
No night shifts (NNS)	Ref	Ref	Ref
No increase in night shifts (NINS)	-2.88 (-6.02 - 0.27)	-1.73 (-3.74 - 0.28)	1.04 (-1.45 - 3.52)
Increase in night shifts (INS)	-4.71 (-7.95 - -1.47)	-1.25 (-3.32 - 0.81)	0.67 (-1.92 - 3.26)

[a] mean difference compared to the reference category, from linear regression models including age, sex, having worked in a COVID-19 ward, the other exposure variable, and the levels of the corresponding burnout dimension in 2019 (Model 3 in the main text).

[b] from Wald chi-square test with 2 degrees of freedom assessing the null hypothesis of no association between the exposure variable and the relevant burnout dimension.
